# Pregnant women as a sentinel population for genomic surveillance of malaria in the Democratic Republic of the Congo: a population-based study

**DOI:** 10.1016/S2214-109X(24)00497-2

**Published:** 2025-02-26

**Authors:** Marie Onyamboko, Varanya Wasakul, Sarah Benie Bakomba, Daddy Kalala Kayembe, Bejos Kifakiou Nzambiwishe, Pascal Epe Ekombolo, Benjamen Basara Badjanga, Jean-Robert Moke Maindombe, Jephte Ndundu Ngavuka, Brunette Nsunda Lwadi, Eleanor Drury, Cristina Ariani, Sonia Goncalves, Vanapol Chamsukhee, Naomi Waithira, Tess D Verschuuren, Sue J Lee, Olivo Miotto, Caterina Fanello

**Affiliations:** a Kinshasa-Oxford Medical Research Unit, Kinshasa School of Public Health, University of Kinshasa, Kinshasa, Democratic Republic of the Congo; b Mahidol-Oxford Tropical Medicine Research Unit (MORU), Faculty of Tropical Medicine, Mahidol University, Bangkok, Thailand; c Wellcome Sanger Institute, Cambridge, UK; d Centre for Tropical Medicine and Global Health, Nuffield Department of Medicine, University of Oxford, Oxford, UK; e Department of Infectious Diseases, The Alfred Hospital and School of Translational Medicine, Monash University, Melbourne, VIC, Australia

## Abstract

**Background:**

Genomic surveillance is a valuable tool for detecting changes in the drug susceptibility of malaria parasites, enabling timely adjustments to treatment strategies. However, implementation can be costly and challenging in high-burden countries, especially when targeting cohorts of children. To address these challenges, we investigated whether in the Democratic Republic of the Congo pregnant women attending antenatal care services could act as an effective sentinel population for children in the same area.

**Methods:**

This population-based study aimed to target pregnant women in Kinshasa (Democratic Republic of the Congo), regardless of age, trimester of pregnancy, parity, or previous antenatal care centre attendance, and children younger than 14 years living in the same area. Women were invited to participate and enrolled during their routine antenatal care visit. For children, we originally planned to conduct standard school-based surveys, but implementation was affected by the COVID-19 pandemic and subsequent vaccination campaign. Therefore, we adopted an alternative approach, setting up screening posts in existing health centres and, with the support of community health workers, encouraging families to visit the posts at their convenience. The study was done in two areas of Kinshasa, urban (Binza) and semirural (Maluku), where malaria transmission is endemic and perennial. Blood samples from malaria-positive cases were genotyped using an amplicon sequencing platform, to allow comparisons of *Plasmodium falciparum* genomes between the two cohorts and estimations of drug resistance mutation prevalence. The study is registered with ClinicalTrials.gov, NCT05072613.

**Findings:**

Between Nov 11, 2021, and June 21, 2023, 2794 children and 4001 pregnant women were recruited to the study. Malaria prevalence by rapid diagnostic test was 49·0% (95% CI 47·1–50·8) in children and 19·1% (17·9–20·3) in pregnant women. Parasite populations sampled from the two cohorts showed highly similar allele frequencies at all tested loci, including drug resistance markers potentially under selection. Pregnant women did not have higher frequencies of sulfadoxine–pyrimethamine resistant haplotypes, which undermine preventive treatments, than children and we did not find any *kelch13* mutations at significant frequency. Although parasite densities were lower in pregnant women, the complexity of infection was similar to that in children. We found no evidence of *Plasmodium vivax* infections in the study.

**Interpretation:**

A cohort of pregnant women produced highly similar results for antimalarial drug resistance surveillance as a cohort of children from the same area, through implementation of simple and efficient genomic surveillance systems integrated into routine antenatal care activities, while benefiting women with diagnosis and treatment.

**Funding:**

Bill & Melinda Gates Foundation and Wellcome Trust.

## Introduction

Drug resistance represents one of the major obstacles to the control and elimination of malaria. The emergence in sub-Saharan Africa of *Plasmodium falciparum* parasites resistant to artemisinin is of particular concern, as this drug is the key component of artemisinin combination therapies, which are used as first-line therapies in all countries in the region.^1^

Large-scale genomic surveillance of *P falciparum* has been done for several years in southeast Asia, where artemisinin-resistant parasites have been circulating for more than a decade, and has proven a valuable tool for mapping resistant strains, detecting changes in antimalarial drug efficacy, and informing public health authorities to allow rapid modification of preventive and curative treatment strategies.^2^, ^3^ The results of genomic surveillance complement those of more costly and complex in vivo therapeutic efficacy studies, which must be done in a more targeted manner and are limited to observing the overall efficacy of an artemisinin combination therapy. Conversely, genomic surveillance can simultaneously detect markers of resistance to individual artemisinin combination therapies and multiple other drugs. Moreover, data can inform on changes in the dynamics of the parasite population, for example, in response to vaccine deployment.

**Research in context**

**Evidence before this study**

Genomic surveillance is a valuable tool for national malaria control programmes to map drug-resistant parasites, but deployment remains challenging in complex, low-resource settings. Given the logistical constraints of targeting young children for routine epidemiological studies, there is growing interest in targeting pregnant women attending antenatal care services as a sentinel population for malaria surveillance. We searched PubMed, with no date or language restrictions, with the search terms “antenatal care”, “malaria”, “surveillance”, “drug-resistant”, “genomic surveillance”, and “Africa”. Search results showed that approaches targeting antenatal care attendees have been tested in Africa, including the Democratic Republic of the Congo, Mozambique, and Tanzania, to measure epidemiological parameters such as disease prevalence. In Mozambique, genomic data from pregnant women were also used to detect changes in transmission and antimalarial drug resistance. The present study tested whether this sampling methodology could be used for genomic surveillance of drug-resistant malaria parasites in an endemic area of the Democratic Republic of the Congo—ie, whether the genomic profile of parasites collected from pregnant women attending antenatal care was similar to that of children living in the same area.

**Added value of this study**

Our results support that pregnant women attending prenatal care in a malaria-endemic setting are an effective sentinel population for genomic surveillance of antimalarial drug resistance. This approach yields results similar to those observed in children living in the same areas, with fewer limitations and additional health-care benefits to participants. Additionally, the study findings were compared with historical data from the same area to provide an overview of resistance trends in the Kinshasa area over time.

**Implications of all the available evidence**

This study expands our knowledge on the use of this survey methodology in Africa, showing its feasibility, informativeness, and direct benefits to participants. In challenging settings with a high malaria burden, passive screening of an adult population for genomic surveillance proved to be a simple and efficient process that can be integrated into routine antenatal care. This approach can inform malaria prevention and treatment strategies for both high-risk groups and the broader population.

The Democratic Republic of the Congo is in the early stages of implementing genomic surveillance for malaria. The country is one of the most affected by the disease, with an estimated 31 million cases and 12% of global malaria deaths,^4^ reflecting the complexity of implementing control activities over a vast territory characterised by institutional and social fragility and ongoing conflict. Because of the difficulties of implementing conventional surveillance methods based on household or school surveys targeting children,^5^ the most affected group in endemic areas, there is growing interest in targeting pregnant women attending antenatal care services as a sentinel population, as this approach presents fewer limitations.^6^, ^7^, ^8^, ^9^, ^10^, ^11^, ^12^, ^13^ In stable high transmission areas, such as most of the Democratic Republic of the Congo, pregnant women are generally semi-immune to malaria, and asymptomatic low-density infections are frequent. The absence of symptoms means that many episodes are not diagnosed, allowing infections to persist over time, with an increased risk of maternal anaemia and adverse pregnancy outcomes due to placental damage. Although standard rapid diagnostic tests can miss low parasitaemia, testing for malaria infections at antenatal care visits is beneficial to mothers and their babies, while providing an opportunity to apply cost-effective sampling protocols for supporting surveillance.

In this study, we aimed to assess whether pregnant women attending antenatal care services in an endemic area are a suitable sentinel population for genomic surveillance of antimalarial drug resistance, and if samples collected from women can provide genomic surveillance results consistent with those in children from the same area. We present a detailed analysis of *P falciparum* genomes from samples collected in two cohorts in Kinshasa, Democratic Republic of the Congo, over a period of 2 years. In addition to a wide range of drug resistance-associated variants, our analysis compared allele frequencies at 100 loci free of selective pressures to check for substantial differences in parasite populations. We also provide a report on drug-resistance mutation frequencies, which we compared with those of studies done previously in Kinshasa.

## Methods

### Study design and population

This population-based study aimed to target pregnant women, regardless of age, trimester of pregnancy, parity, or previous antenatal care centre attendance, and children younger than 14 years living in the same area. Women were invited to participate and enrolled during their routine antenatal care visit. For children, we originally planned to conduct standard school-based surveys, but implementation was affected by the COVID-19 pandemic and subsequent vaccination campaign. Therefore, we adopted an alternative approach, setting up screening posts in existing health centres and, with the support of community health workers, encouraging families to visit the posts at their convenience. All study procedures were done by trained research personnel. Each participant was included in the study once. The study was done in two areas of Kinshasa, urban (Binza) and semirural (Maluku), where malaria transmission is endemic and perennial.^14^

Our research adhered to the ethical principles outlined in the Declaration of Helsinki for medical studies involving human subjects. All participants (or their parents or guardians in the case of minors) provided written consent after a comprehensive explanation given in their preferred language before enrolment in the study. Approval was given by the research ethics committee of the University of Oxford (OxTREC 548-21), the Kinshasa School of Public Health (ESP/CE133/2021), and the Ministry of Health of the Democratic Republic of the Congo (025/GPK/CAB/MIN.SPH/LNK/NIP/ski/2021). Further approval was granted for screening schoolchildren by the Provincial Ministry of Health, the Provincial Ministry of Education, the Provincial Division Head, and the National Intelligence Agency. Approval for exporting blood samples was granted by the Ministry of the Environment and Sustainable Development in accordance with the Nagoya Protocol. The study is registered with ClinicalTrials.gov, NCT05072613.

### Procedures

Participants were tested for malaria with a *pfhrp2*-based rapid diagnostic test (SD Bioline *Malaria* Ag Pf/Pan; Standard Diagnostics; Yongin-si, South Korea) and haemoglobin levels were measured with HemoCue 301 (HemoCue; Angelholm, Sweden). A capillary blood sample was taken from each patient with a positive result on rapid diagnostic test to determine parasite species and density by standard microscopy and to prepare a dried blood spot (DBS) for genomic analysis. Individuals with malaria or anaemia were offered treatment as per national guidelines in the Democratic Republic of the Congo or were referred to hospital if severe. Demographic and anthropometric data were collected from all participants. Epidemiological data were entered locally using the REDCap electronic data capture tool hosted by the University of Oxford.

DBSs were processed by the Wellcome Sanger Institute by use of the SpotMalaria *Plasmodium* genotyping platform.^2^ Results were obtained for samples from 644 pregnant women (84% of the 766 DBSs collected) and 1356 children (99% of the 1368 DBSs collected). Data were encoded into a Genetic Report Card format, comprising genotypes at drug resistance-related single nucleotide polymorphisms (SNPs), as well as genetic barcodes. The genetic barcode consisted of 100 biallelic SNPs distributed across the nuclear chromosomes, known to be variable globally and not related to drug resistance.^2^

In any given sample, SNPs with insufficient read coverage to call a genotype were encoded as missing, whereas SNPs with reads for both alleles were encoded as heterozygous. Sample missingness (a measure of genotyping quality) was estimated as the proportion of barcoding SNPs encoded as missing, whereas sample heterozygosity was estimated as the proportion of non-missing barcoding SNPs encoded as heterozygous and used to estimate complexity of infection. Samples with more than 50% missingness were deemed to be of low genotyping quality and excluded from heterozygosity analyses. At any given SNP, the non-reference allele frequency (NRAF) in a population was determined by excluding samples with missing and heterozygous encoding, and then determining the proportion of the remaining samples that carried the non-reference allele. Infecting *Plasmodium* species were identified by kraken2 (version 2.0.7)^15^ and summarised with krakentools (version 1.2), using a custom database comprising mitochondrial sequences of six *Plasmodium* species (appendix 1 p 3). We deemed a species to be present if the classifier assigned at least five reads and at least 1% of reads for that sample. To analyse changes in drug-resistant allele frequencies over time, we compared frequencies in children with earlier data from clinical therapeutic efficacy trials conducted in the paediatric population between 2012 and 2016 in Kinshasa and available from the MalariaGEN Pf7 dataset.^16^

### Statistical analysis

A sample size of 650 DBSs from each cohort was estimated to enable accurate estimation of the prevalence (up to 50% ±4%) of antimalarial drug resistance-associated mutations with 95% confidence. Since malaria prevalence in this area ranges from 15% to 30%,^17^ we aimed to screen a minimum of 3540 individuals in each cohort. Comparisons used the χ^2^ or Kruskal-Wallis test and correlations were assessed using Kendall's τ. Parasite densities were reported as geometric mean with 95% CIs and correlations assessed using non-parametric methods. Allele frequencies were compared using a z-test with Agresti-Coull 95% CIs for proportions (BinomCI; DescTools R package version 0.99.54^18^). Logistic regression with presence of alleles or genotypes as the dependent variable was used to determine association with cohort. Since the proportions of women and children differed between urban and semirural areas, all models included adjustment for area. Data were analysed using STATA version 18.0 and R version 4.2.3.

### Role of the funding source

The funders of the study had no role in study design, data collection, data analysis, data interpretation, or writing of the report.

## Results

Between Nov 11, 2021, and June 21, 2023, 2794 children and 4001 pregnant women were recruited to the study (appendix 1 pp 5, 6). Malaria prevalence by rapid diagnostic test was 49·0% (95% CI 47·1–50·8) in children and 19·1% (17·9–20·3) in pregnant women (table 1). 188 (13·7%) of 1368 rapid diagnostic test-positive cases among children and 62 (8·1%) of 766 among women were negative by microscopy, with a larger difference observed in children (p<0·0001), probably due to a higher number of recently treated infections with detectable *Pf*HRP2 antigen levels in children than in pregnant women. Within each cohort, parasite densities correlated negatively with age (Kendall's τ –0·057, p=0·0030 in children; –0·159, p<0·0001 in women). Women had similar prevalences of malaria in urban and semirural area (p=0·23), whereas parasitaemia was higher in semirural areas, likely because participants were on average younger and more like to be primiparous (appendix 1 p 4). Children in semirural areas were significantly more likely to have malaria and have higher parasite levels than those in urban areas (p<0·0001; appendix 1 p 4). This finding could be partly explained by a higher proportion of sick children presenting at the health-care facility in the semirural areas, since this clinic routinely provides free malaria screening. The final sample size was larger than planned, to account for the high prevalence of malaria in the child cohort, the need to sample both cohorts simultaneously, and the discrepancy observed between microscopy and rapid diagnostic test results.

**Table 1:** Summary of demographic, anthropometric, and laboratory results in the two cohorts (children *vs* pregnant women)

		**Children (n=2794)**	**Pregnant women (n=4001)**
Place of enrolment			
	School	92 (3·3%)	..
	Health centre	2702 (96·7%)	..
	Antenatal care service	..	4001 (100%)
Location			
	Urban	892 (31·9%)	3057 (76·4%)
	Semirural	1902 (68·0%)	944 (23·6%)
Sex			
	Female	1388 (49·7%)	4001 (100%)
	Male	1406 (50·3%)	..
Age, years		6·1 (3·0–9·4)	28·7 (24·0–33·4)
Date of birth unknown		18 (0·6%)	39 (1·0%)
Pregnant teenagers (aged <19 years)		..	220 (5·5%)
Primiparae		..	758 (18·9%)
Body temperature, °C		36·5 (36·2–36·8)	36·3 (36·2–36·6)
Fever (>37·5°C)		215 (7·7%)	7 (0·2%)
Report of sickness in the previous days		1989 (71·2%)	..
Positive on rapid diagnostic test (*P falciparum* or pan-specific)		1368 (49·0%)*	765 (19·1%)
*P falciparum* by microscopy		1180/1368 (86·3%)	703/765 (91·9%)
Geometric mean parasitaemia (95% CI), μL		5998 (5031–7150)	571 (486–672)
Hyperparasitaemia (>100 000 copies per μL)		236/1180 (20·0%)	3/703 (0·4%)
Dried blood spots with available results		1356	644

Data are n (%), median (IQR), n/N (%), or n, unless otherwise indicated. *P falciparum*=*Plasmodium falciparum*.

* In five cases only the pan-specific band was present; all five cases showed a *P falciparum* mono-infection by microscopy.

We could identify the infecting species in 1967 (98·4%) of 2000 samples analysed, all of which contained *P falciparum* parasites. Co-infections with *Plasmodium malariae* were detected in 160 samples (8·0 % in children and 8·3% in women) and with *Plasmodium ovale* in 56 samples (3·2% in children and 2·2% in women; p=0·70); both species were found in varying proportions within samples (1–99% of sequencing reads). We found no evidence of *Plasmodium vivax* and *Plasmodium knowlesi*.

**Figure 1:**
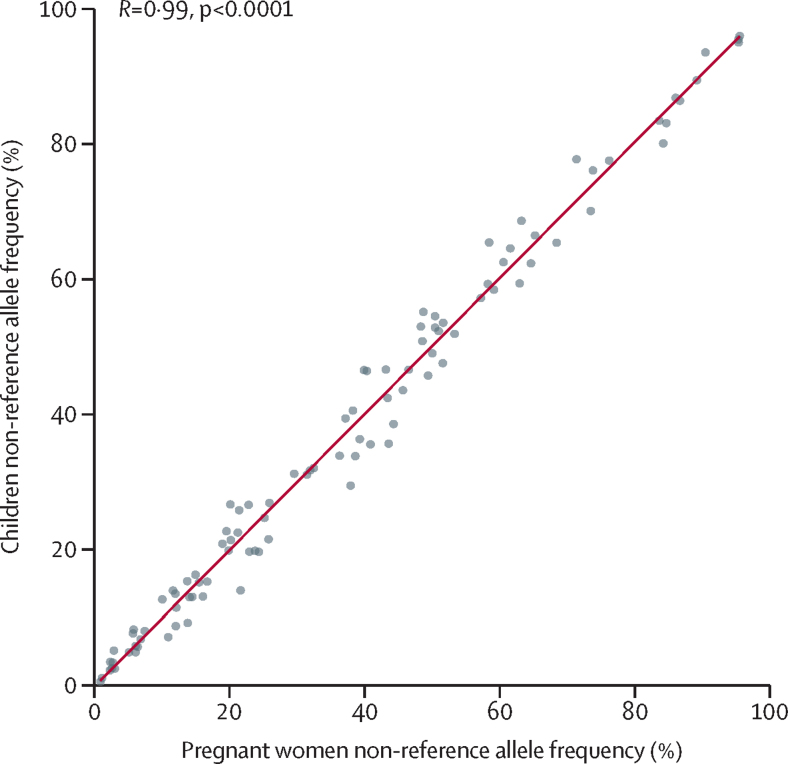
Non-reference allele frequency correlation between cohorts Non-reference allele frequencies were estimated at each of the 100 biallelic single nucleotide polymorphism barcodes.

We found strong positive correlation between the NRAF in the two cohorts (figure 1; r=0·99; p<0·0001). Only two variants showed significant differences between cohorts (barcoding SNP 95 p=0·0009 and SNP 37 p=0·0068) but in both cases the differences were moderate (0·22, 95% CI 0·18–0·26 in women *vs* 0·14, 0·12–0·16 at SNP 95 in children; 0·38, 0·33–0·43 in women *vs* 0·29, 0·26–0·33 at SNP 37 in children; appendix 1 p 7). At all remaining SNPs, confidence intervals of the frequency estimates overlapped.

We estimated allele frequencies at several known drug resistance loci that might be under selection pressure in the Democratic Republic of the Congo (table 2). Allele differences at these loci suggest that parasites infecting the two cohorts are subject to different selective pressures.

**Table 2:** Comparison of allele frequencies in the two cohorts at SNPs associated with antimalarial drug resistance

	**Chromosome and position**	**Reference allele**	**Non-reference allele**	**Non-reference allele frequency in children**	**Non-reference allele frequency in pregnant women**	p value*
*dhfr*	51	N	I	1107/1113 (99·5%)	499/503 (99·2%)	0·84
*dhfr*	59	C	R	932/1004 (92·8%)	430/463 (92·9%)	0·82
*dhfr*	108	S	N	1097/1103 (99·5%)	475/478 (99·4%)	0·66
*dhfr*	164	I	L	0	0	1·00
*dhps*	436	S	A	74/1014 (7·3%)	40/433 (9·2%)	0·021
*dhps*	437	A	G	1038/1067 (97·3%)	435/444 (98·0%)	0·46
*dhps*	540	K	E	185/889 (20·8%)	59/339 (17·4%)	0·92
*dhps*	581	A	G	112/921 (12·2%)	40/348 (11·5%)	0·57
*dhps*	613	A	S	34/993 (3·4%)	17/373 (4·6%)	0·035
*crt*	74	M	I	131/1016 (12·9%)	36/448 (8·0%)	0·11
*crt*	75	N	E	129/1011 (12·8%)	37/447 (8·3%)	0·15
*crt*	76	K	T	132/1016 (13·0%)	39/453 (8·6%)	0·16
*crt*	220	A	S	157/959 (16·4%)	49/456 (10·7%)	0·069
*crt*	271	Q	E	143/969 (14·8%)	41/451 (9·1%)	0·075
*crt*	356	T	I	104/1009 (10·3%)	40/450 (8·9%)	0·89
*crt*	371	R	I	156/976 (16·0%)	49/465 (10·5%)	0·16
*mdr1*	86	N	Y	54/1030 (5·2%)	27/431 (6·3%)	0·23
*mdr1*	184	Y	F	226/798 (28·3%)	103/350 (29·4%)	0·21
*mdr1*	1246	D	Y	5/1011 (0·5%)	0	0·99

Data are n/N (%), unless otherwise indicated. The table shows allele frequencies at positions that were tested for known mutations associated with drug resistance. SNP=single nucleotide polymorphism. The last column indicates, for *dhfr* and *dhps* SNPs only, the sulfadoxine–pyrimethamine resistant haplotype that includes the mutation.

* p value of logistic regression adjusted by area to determine whether the mutation prevalence was significantly different in the two cohorts.

We were able to fully scan the kelch and BTB/POZ domains of the *kelch13* gene (PF3D7_1343700)^19^ in 1316 samples but did not find any mutation associated with artemisinin resistance at elevated frequency. Besides the Ala578Ser variant, which has been shown not to confer artemisinin resistance,^20^ only the Val534Ala mutation, observed in two cases, was identified as a homozygous variant. Six other mutations (Pro527His, Ser522Cys, Gly533Ser, Gln613Glu, Ile634Leu, and Asp641Asn) were each found in a single mixed infection. The low frequency of these mutations, and their presence in mixed infections, is consistent with the expected prevalence of these variants in African countries under normal conditions.^21^

We found minimal or non-significant differences between cohorts in variants within the *dhfr* (PF3D7_0417200) and *dhps* (PF3D7_0810800) genes associated with antifolate resistance (table 2). We observed several combinations of *dhfr* and *dhps* mutations that have been associated with reduced parasite sensitivity to sulfadoxine–pyrimethamine. The triple mutant haplotype (comprising *dhfr* Asn51Ile, Cys59Arg, and Ser108Asn), which confers resistance to pyrimethamine,^22^ was found in 1267 (92·5%) of 1370 isolates, whereas the quintuple mutant haplotype (triple mutant with additional mutations Ala437Gly and Lys540Glu in *dhps*), associated with failure to sulfadoxine–pyrimethamine,^22^ was detected in 213 (20·2%) of 1055 isolates (table 3). The sextuple mutant haplotype (carrying the additional *dhps* Ala581Gly mutation) that further reduces sulfadoxine–pyrimethamine sensitivity and compromises the efficacy of intermittent preventive treatment in pregnancy,^22^ was detected in 88 (9·2%) of 962 samples. Furthermore, the *dhps* Ala581Gly mutation was detected as part of a sextuple haplotype and, in about a third of samples, in isolation. The *dhps* Ala613Ser mutation, an alternative sextuple haplotype variant, was detected in 51 samples, but only in isolation. The *dhfr* Ile164Leu mutation, also a potential sextuple mutant component, was absent in our dataset, consistent with its low prevalence in Africa.^23^

**Table 3:** Comparison of sulfadoxine–pyrimethamine-resistant haplotypes in the two cohorts

	**Resistant haplotype**	**Resistant haplotype frequencies in children**	**Resistant haplotype frequencies in pregnant women**	p value*
Triple	IRN	884/955 (92·6%)	383/415 (92·3%)	0·74
Quintuple	IRNGE	167/766 (21·8%)	46/289 (15·9%)	0·61
Sextuple	IRNGEG	69/704 (9·8%)	19/258 (7·4%)	0·42

Data are n/N (%), unless otherwise indicated. This table shows the frequencies of haplotypes resistant to sulfadoxine–pyrimethamine. Each row represents a haplotype. Only one form of the sextuple mutant (IRNGEG) was identified in the present study.

* p value of the logistic regression to determine whether the prevalence of the haplotype was significantly different in the two cohorts.

The analysis of polymorphisms in the *crt* gene (PF3D7_0709000), which is associated with resistance to chloroquine and amodiaquine,^24^, ^25^ also did not reveal significant differences between cohorts. The most common chloroquine-resistant haplotype (often referred to as CVIET for its amino acids at positions 72–76) was detected in 129 (12·8%) of 1009 children and 36 (8·1%) of 444 pregnant women (p=0·13 adjusted by area). The *crt* gene mutations associated with piperaquine resistance in southeast Asia (eg, at positions 97, 218, and 333) were absent from our sample set. Mutations in the *mdr1* gene (PF3D7_0523000) were generally at low levels, except for the Tyr184Phe mutation, which was present in 329 (28·7%) of 1148 samples, without significant differences between cohorts.

**Figure 2:**
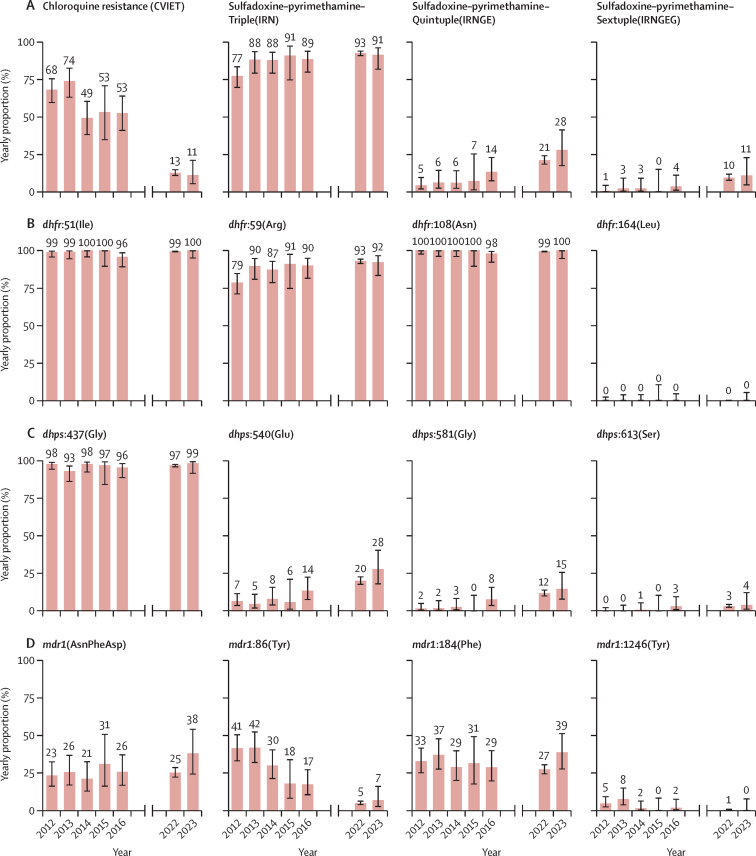
Temporal trends of drug-resistant haplotypes and alleles in children from Kinshasa Values represent the yearly proportion of haplotypes conferring chloroquine (PfCRT) and sulfadoxine–pyrimethamine resistance (triple, quintuple, and sextuple; A), *dhfr* alleles (B), *dhps* alleles (C), and *mdr1* haplotype and alleles (D). 15 samples, collected towards the end of 2021, were aggregated with data from 2022. Error bars indicate 95% CIs.

A comparison of current allele frequencies at drug-resistant loci in *crt*, *dhps, dhfr*, and *mdr1* against 573 samples collected in 2012–16 showed major changes in drug resistance profiles (figure 2). The frequency of the CVIET haplotype decreased significantly, from 61·6% in 2012–16 to 12·8% in 2021–23 (p<0·0001). Similarly, the mutation at position Asn86Tyr on *mdr1* decreased significantly from 32·2% to 5·2% (p<0·0001). By contrast, no differences were found in the frequency of the Tyr184Phe mutation. This analysis showed a substantial increase in the prevalence of all haplotypes conferring resistance to sulfadoxine–pyrimethamine, largely driven by a significant increase in the frequencies of the *dhps* Lys540Glu and Ala581Gly mutations (540Glu from 7·9% to 20·8%; p<0·0001; 581Gly from 3·0% to 12·2%; p<0·0001).

In this study, we observed a strong negative correlation between parasite densities and genotype missingness in both cohorts (appendix 1 p 8; Kendall's τ –0·437 in women and –0·510 in children; both p<0·0001). As a result of low parasitaemia, 26·4% (95% CI 23·1 to 29·9%) of samples in women and 17·6% (15·7 to 19·7) of samples in children had more than 50% missingness, suggesting that applying a minimum parasitaemia threshold might increase surveillance cost efficiency, and that the antenatal care population might produce somewhat smaller sample sizes for certain types of analyses.

We used the heterozygosity level, defined here as the proportion of non-missing barcode SNPs assigned a heterozygous genotype, as a measure of the complexity of infection. Heterozygosity levels correlate well with complexity of infection estimates predicted by the RealMcCoil bioinformatic tool.^26^ Complexity of infection estimates ranged from 1 to 5, with approximately 50% of infections being polyclonal (complexity of infection ≥2) in both cohorts. The median heterozygosity level was 0·16 (IQR 0·01–0·35), and although children had slightly higher values than women (0·17, 0·10–0·37 *vs* 0·14, 0·02–0·30; p=0·15; appendix 1 p 7), age and heterozygosity were not correlated (Kendall's τ –0·015; p=0·37).

## Discussion

Genomic surveillance has proven itself a very useful tool for informing malaria control programmes tracking the emergence and spread of antimalarial drug-resistant strains. Yet, the most fragile countries, which are also those with the highest burden of disease and thus could benefit most from the availability of new tools, are also those with the greatest difficulties in terms of implementation. There is an imperative to find strategies that can simplify deployment of genomic surveillance and seamless integration into public health processes.

In this study, we took a step towards overcoming some key implementation issues by testing a pragmatic approach that is simple to integrate into routine public health operations. We established that a cohort of pregnant women in the Democratic Republic of the Congo, passively screened at antenatal care, produced the same genomic surveillance results as a cohort of children, which would be more difficult to monitor.

Our population included women of all ages, trimesters, and parities, reflecting the real-life composition of a population of antenatal care attendees. Participants were screened using standard rapid diagnostic tests, which cannot measure true disease prevalence, but are readily available, inexpensive, and sufficiently sensitive for our platform. Implementing genomic surveillance at antenatal care centres is simpler and more economical than using other cohorts, and complements existing prevention strategies by strengthening diagnosis in asymptomatic women. Antenatal care services are available in most of the Democratic Republic of the Congo and approximately 80% of women visit at least once during pregnancy.^27^ Although coverage is uneven and below desirable levels, it is sufficient to establish genomic surveillance in most provinces.

An important finding from this study is that allele frequencies estimated from women were similar to those from children, not only at neutral barcoding SNPs, but also for mutations associated with resistance to antimalarial drugs. This finding was by no means a given; since women and children are not exposed to the same antimalarial drugs and show different age-related responses to infection, differences could not be ruled out. Notably, the two parasite populations had several other similar characteristics. For example, levels of complexity of infection, which result from bites of mosquitoes infected with more than one clone or multiple bites, were similar in both cohorts, even though women tend to harbour longer-lasting asymptomatic parasite reservoirs. These pronounced levels of heterozygosity suggest that pregnant women can also provide valuable information about changes in transmission intensity^28^ (eg, in response to vaccine deployment).

In the process of showing the equivalence of the two cohorts, we also estimated current levels of drug-resistant mutation prevalence in Kinshasa and described their trends for the last decade. Reassuringly, we found no evidence of artemisinin resistance mutations in *kelch13*, which are increasingly being reported in east Africa^1^ and the eastern borders of the Democratic Republic of the Congo;^29^ although geographically distant from the areas affected, Kinshasa is a recipient for internally displaced people from other provinces, particularly from the eastern Democratic Republic of the Congo, and could become a spreading hub.

Resistance to antifolates poses a more pressing problem because of widespread resistant haplotypes in the *dhfr* and *dhps* genes, which undermine chemopreventive sulfadoxine–pyrimethamine treatment. We found more than 90% of infections harbouring triple mutant haplotypes, 20% carrying quintuple, fully resistant haplotypes, and about 10% with the sextuple super-resistant haplotype. Two mutations normally considered part of sextuple mutant haplotypes (*dhps* Ala613Ser/Thr and Ala581Gly) were frequently found without the remainder of the haplotypes, highlighting the marked variability of the *dhps* gene.^30^, ^31^ The frequency of resistant haplotypes and the rise in frequency of some mutations over time suggest continued pharmacological selective pressure from sulfadoxine–pyrimethamine, which in the Democratic Republic of the Congo is mainly used for intermittent preventive treatment in pregnancy. Although the benefits of intermittent preventive treatment with sulfadoxine–pyrimethamine can still outweigh the risks of adverse birth outcomes, results suggest that, if the trend towards loss of efficacy continues, the country should evaluate chemopreventive alternatives. Dihydroartemisinin–piperaquine has been shown to significantly improve maternal health compared with sulfadoxine–pyrimethamine,^32^ with a good safety profile in pregnancy as well as in HIV-positive women.^32^, ^33^

Analyses of historical trends also showed that resistance to the 4-aminoquinoline chloroquine, mediated by mutations in the *crt* gene, was low, with evidence of a temporal decline over the past decade. This downward trend was accompanied by a decline in the frequency of the Asn86Tyr mutation on the *mdr1* gene. The role of this mutation is unclear, as it has been associated with reduced sensitivity to amodiaquine (another 4-aminoquinoline), but there is some evidence that lumefantrine might select for the wild-type allele.^34^ Since amodiaquine-artesunate and artemether-lumefantrine are both part of a first-line multitherapy strategy in the Democratic Republic of the Congo, and are both available on the market, the *mdr1* Asn86Tyr trend remains difficult to interpret. Reliable, validated genetic markers for resistance to amodiaquine and lumefantrine are urgently needed if critically needed monitoring of artemisinin combination therapy partner drug efficacy in Africa is to be supported.

The use of an antenatal centre sentinel population has some drawbacks that should be considered. Although about 20% of women were found to be infected, providing an abundant supply of samples, the lower parasite densities translated to lower parasite DNA content, and therefore a higher chance of genotyping failure. To avoid wastage, genomic surveillance studies should establish a minimum parasitaemia threshold and screen samples accordingly. Arguably, cohorts of school children might also have high proportions of low-parasitaemia infections, since sick children might be absent from school and many disadvantaged children, who are most at risk of being infected, often do not attend school.

Pragmatic implementation strategies, such as the one we recommend here, are only enabling components of complex implementations. There are other systemic obstacles to implementing genomic surveillance in low-resource settings, such as difficulties in building and maintaining local technical capacities and costs and delays associated with procurement of consumables. Suitable strategic choices can only succeed with donor support, alignment with ongoing local capacity-building efforts, and input from the scientific community.

In conclusion, our results extend existing knowledge of the characteristics of antenatal care attendees as a sentinel population and show their potential for implementation of genomic surveillance of antimalarial drug resistance. The advantages are substantial, as this model of surveillance can be applied to other pathogens and antimicrobial resistance, providing direct benefits to participants.

### Contributors

### Data sharing

De-identified participant data are available from the Mahidol Oxford Tropical Medicine Data Access Committee upon request from this link: https://www.tropmedres.ac/units/moru-bangkok/bioethics-engagement/datasharing. Parasite genetic data for 2000 analysed cases are available in tabular form in appendix 2.

## Declaration of interests

We declare no competing interests.

## References

[1] Rosenthal PJ, Asua V, Conrad MD (2024). Emergence, transmission dynamics and mechanisms of artemisinin partial resistance in malaria parasites in Africa. Nat Rev Microbiol.

[2] Jacob CG, Thuy-Nhien N, Mayxay M (2021). Genetic surveillance in the Greater Mekong subregion and south Asia to support malaria control and elimination. eLife.

[3] Noviyanti R, Miotto O, Barry A (2020). Implementing parasite genotyping into national surveillance frameworks: feedback from control programmes and researchers in the Asia-Pacific region. Malar J.

[4] WHO (2023).

[5] Cibulskis RE, Bell D, Christophel EM (2007). Estimating trends in the burden of malaria at country level. Am J Trop Med Hyg.

[6] van Eijk AM, Hill J, Noor AM, Snow RW, ter Kuile FO (2015). Prevalence of malaria infection in pregnant women compared with children for tracking malaria transmission in sub-Saharan Africa: a systematic review and meta-analysis. Lancet Glob Health.

[7] Willilo RA, Molteni F, Mandike R (2016). Pregnant women and infants as sentinel populations to monitor prevalence of malaria: results of pilot study in Lake Zone of Tanzania. Malar J.

[8] Hellewell J, Walker P, Ghani A, Rao B, Churcher TS (2018). Using ante-natal clinic prevalence data to monitor temporal changes in malaria incidence in a humanitarian setting in the Democratic Republic of Congo. Malar J.

[9] Brunner NC, Chacky F, Mandike R (2019). The potential of pregnant women as a sentinel population for malaria surveillance. Malar J.

[10] Kitojo C, Gutman JR, Chacky F (2019). Estimating malaria burden among pregnant women using data from antenatal care centres in Tanzania: a population-based study. Lancet Glob Health.

[11] Mayor A, Menéndez C, Walker PGT (2019). Targeting pregnant women for malaria surveillance. Trends Parasitol.

[12] Brokhattingen N, Matambisso G, da Silva C (2024). Genomic malaria surveillance of antenatal care users detects reduced transmission following elimination interventions in Mozambique. Nat Commun.

[13] Gutman JR, Thwing J, Mwesigwa J, McElroy PD, Robertson M (2022). Routine healthcare facility- and antenatal care-based malaria surveillance: challenges and opportunities. Am J Trop Med Hyg.

[14] Ferrari G, Ntuku HM, Schmidlin S, Diboulo E, Tshefu AK, Lengeler C (2016). A malaria risk map of Kinshasa, Democratic Republic of Congo. Malar J.

[15] Wood DE, Lu J, Langmead B (2019). Improved metagenomic analysis with Kraken 2. Genome Biol.

[16] Ahouidi A, Ali M, Almagro-Garcia J (2021). An open dataset of *Plasmodium falciparum* genome variation in 7000 worldwide samples. Wellcome Open Res.

[17] Fanello C, Lee SJ, Bancone G (2023). Prevalence and risk factors of neonatal hyperbilirubinemia in a semi-rural area of the Democratic Republic of Congo: a cohort study. Am J Trop Med Hyg.

[18] Signorell A (2024). DescTools: Tools for Descriptive Statistics. https://cran.rstudio.com/web/packages/DescTools/DescTools.pdf.

[19] Ariey F, Witkowski B, Amaratunga C (2014). A molecular marker of artemisinin-resistant *Plasmodium falciparum* malaria. Nature.

[20] WHO (2018).

[21] Miotto O, Amato R, Ashley EA (2015). Genetic architecture of artemisinin-resistant *Plasmodium falciparum*. Nat Genet.

[22] Naidoo I, Roper C (2013). Mapping ‘partially resistant’, ‘fully resistant’, and ‘super resistant’ malaria. Trends Parasitol.

[23] Lynch C, Pearce R, Pota H (2008). Emergence of a dhfr mutation conferring high-level drug resistance in *Plasmodium falciparum* populations from southwest Uganda. J Infect Dis.

[24] Fidock DA, Nomura T, Talley AK (2000). Mutations in the *P falciparum* digestive vacuole transmembrane protein PfCRT and evidence for their role in chloroquine resistance. Mol Cell.

[25] Happi CT, Gbotosho GO, Folarin OA (2006). Association between mutations in *Plasmodium falciparum* chloroquine resistance transporter and *P falciparum* multidrug resistance 1 genes and in vivo amodiaquine resistance in *P falciparum* malaria-infected children in Nigeria. Am J Trop Med Hyg.

[26] Chang HH, Worby CJ, Yeka A (2017). THE REAL McCOIL: a method for the concurrent estimation of the complexity of infection and SNP allele frequency for malaria parasites. PLOS Comput Biol.

[27] UNICEF (2024). UNICEF data: monitoring the situation of children and women. https://data.unicef.org/topic/maternal-health/antenatal-care/.

[28] Schneider KA, Tsoungui Obama HCJ, Kamanga G, Kayanula L, Adil Mahmoud Yousif N (2022). The many definitions of multiplicity of infection. Front Epidemiol.

[29] Mesia Kahunu G, Wellmann Thomsen S, Wellmann Thomsen L (2024). Identification of the PfK13 mutations R561H and P441L in the Democratic Republic of Congo. Int J Infect Dis.

[30] Mita T, Venkatesan M, Ohashi J (2011). Limited geographical origin and global spread of sulfadoxine-resistant dhps alleles in *Plasmodium falciparum* populations. J Infect Dis.

[31] Plowe CV (2022). Malaria chemoprevention and drug resistance: a review of the literature and policy implications. Malar J.

[32] Kajubi R, Ochieng T, Kakuru A (2019). Monthly sulfadoxine-pyrimethamine versus dihydroartemisinin-piperaquine for intermittent preventive treatment of malaria in pregnancy: a double-blind, randomised, controlled, superiority trial. Lancet.

[33] Barsosio HC, Madanitsa M, Ondieki ED (2024). Chemoprevention for malaria with monthly intermittent preventive treatment with dihydroartemisinin-piperaquine in pregnant women living with HIV on daily co-trimoxazole in Kenya and Malawi: a randomised, double-blind, placebo-controlled trial. Lancet.

[34] Sisowath C, Ferreira PE, Bustamante LY (2007). The role of pfmdr1 in *Plasmodium falciparum* tolerance to artemether-lumefantrine in Africa. Trop Med Int Health.

