# Supplementary material for: Pregnant women as a sentinel population for genomic surveillance of malaria in the Democratic Republic of the Congo: a population-based study
Source: Lancet Glob Health. 2025 Feb 26;13(3):e479–87. doi: 10.1016/S2214-109X(24)00497-2 (PMC11868776; doi:10.1016/S2214-109X(24)00497-2)

THE LANCET
Global Health

Supplementary appendix 1

This appendix formed part of the original submission and has been peer reviewed. We post it as supplied by the authors.

Supplement to: Onyamboko M, Wasakul V, Bakomba SB, et al. Pregnant women as a sentinel population for genomic surveillance of malaria in the Democratic Republic of the Congo: a population-based study. *Lancet Glob Health* 2025; **13:** e479–87.

Onyamboko *et al*. Pregnant women as a sentinel population for genomic surveillance of malaria in the Democratic Republic of Congo

Supplementary Materials

Abstract

Introduction La surveillance Génomique est un outil précieux pour détecter les changements de la susceptibilité aux médicaments des parasites de la malaria, permettant des ajustements précoces des stratégies de traitement. Toutefois, son implémentation peut être couteuse et difficile à établir dans les pays fragiles et à forte charge palustre, et particulièrement quand des cohortes d'enfants sont impliquées. Pour relever ces défis, nous avons évalué si en République Démocratique du Congo, les femmes enceintes fréquentant les services de consultations prénatales (CPNs) pouvaient servir de population sentinelle de substitution.

Méthode Entre Novembre 2021 et Juin 2023, nous avons conduit une étude à Kinshasa, ciblant 4,001 femmes enceintes s'étant présentées aux CPNs, et 2,794 enfants vivant dans la même région. Les échantillons de sang provenant des cas positifs pour la malaria ont été génotypé avec une plateforme de séquençage basée sur l'amplicon, permettant la comparaison entre les deux cohortes des génomes des *Plasmodium falciparum* et l'estimation de la prévalence des mutations liées à la résistance médicamenteuse (ClinicalTrials.gov Identifier NCT05072613).

Résultats principaux Les populations parasitaires des deux cohortes ont montré des fréquences d'allèle très similaire à tous les locus testés incluant les marqueurs moléculaires de la résistance médicamenteuses potentiellement sous sélection. Les femmes enceintes n'ont pas présenté des fréquences plus élevées d'haplotypes résistant de sulphadoxine-pyriméthamine qui sapent les traitements préventifs, que celles des enfants, et nous n'avons trouvé aucune fréquence significative de mutation *kelch13*. Bien que les densités parasitaires étaient plus faibles chez les femmes enceintes, la complexité de l'infection était similaire à celle vue chez les enfants. Il n'y a eu aucune évidence d'infection à *Plasmodium vivax* dans l'étude.

Interprétation Une cohorte de femmes enceintes a produit des résultats très similaires à ceux des enfants, permettant de proposer l'implémentation d'un système simple et efficient de surveillance génomique intégré dans les activités de routine des CPNs, bénéficiant aux femmes pour le diagnostic et le traitement.

Financement Cette étude a été financée par la Fondation Bill et Melinda Gates et le Wellcome Trust.

Supplementary Table 1. Location of mitochondrial sequence sources used to classify six *Plasmodium* species

Species	Record Location
P. falciparum **(Pf)**	https://plasmodb.org/plasmo/app/record/genomic-sequence/download/Pf3D7_MIT_v3
P. vivax **(Pv)**	https://plasmodb.org/plasmo/app/downloads/Current_Release/PvivaxSal1 (contig PVAD80_MIT)
P. knowlesi **(Pk)**	https://plasmodb.org/plasmo/app/record/genomic-sequence/download/PKNH_MIT_v2
P. malariae **(Pm)**	https://www.ncbi.nlm.nih.gov/nuccore/LT594637
P. ovale curtisi **(Poc)**	https://www.ncbi.nlm.nih.gov/nuccore/HQ712052.1
P. ovale wallikeri **(Pow)**	https://www.ncbi.nlm.nih.gov/nuccore/HQ712053

Supplementary Table 2. Summary of *P. falciparum* characteristics in the two cohorts by area

Characteristics by cohort	Urban	Semirural	p-value
**Women**			
Total enrolled	3,057	944	
Positive by RDT, N (%)	598 (19·6)	168 (17·8)	0·228
Positive cases by microscopy, N (%)	548 (91·6)	156 (92·9)	0·623
Geo. mean parasitaemia/µL (95% CI)	502 (417-605)	889 (647-1,221)	<0·001
% Primiparae	523 (17·1)	235 (25·2)	
Positive primiparae by RDT N (%)	183 (35·0)	72 (30·6)	0·241
Positive primiparae by microscopy N (%)	548 (91·9)	156 (92·9)	0.609
Geo. mean parasitaemia/µL in primiparae (95% CI)	766 (546-1073)	1,346 (820-2209)	0·044
**Children**			
Total enrolled	892	1,902	
P. falciparum by RDT, N (%)	318 (55·2)	1,050 (35·7)	<0·001
Positive cases by microscopy, N (%)	275 (86·5)	905 (86·2)	0.896
Geo. mean parasitaemia/µL (95% CI)	2,361 (1,641-3,397)	7,961 (6,535-9,699)	<0·001

Supplementary Figure 1. Flow chart of the study (children)

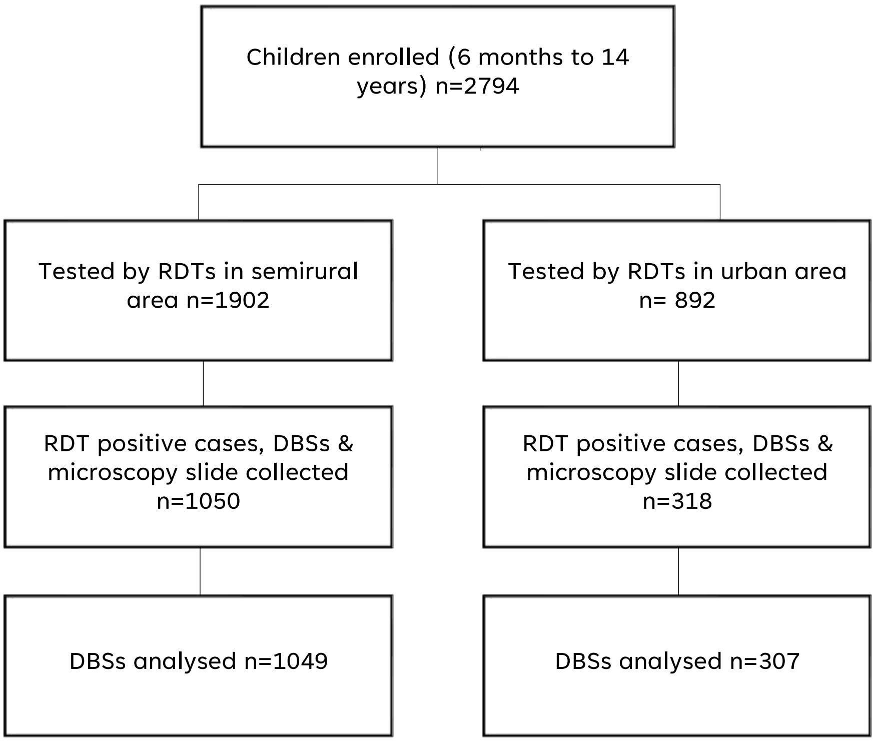

Supplementary Figure 2. Flow chart of the study (pregnant women)

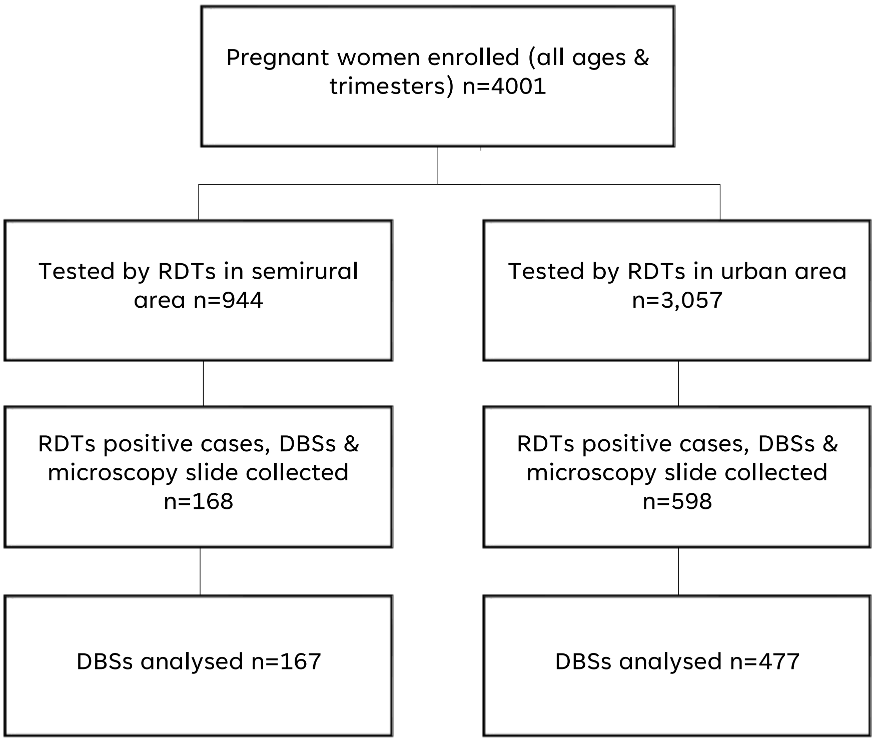

Supplementary Figure 3. Estimated non-reference allele frequencies (NRAF) and 95% confidence intervals at each barcode SNP position in each cohort

Overlapping of confidence intervals of the frequency estimates indicates minimal differentiation in the population between the two cohorts. Pregnant women's NRAF are coloured in blue with 95% confidence intervals shaded in. Children's NRAF are in black with error bars indicating 95% confidence interval. Red colour highlights children's NRAF where there are non-overlapping confidence intervals between the two cohorts (Barcode SNP #95 and #37). To improve visualisation, barcode SNP positions are arranged in ascending order of pregnant women's NRAF.

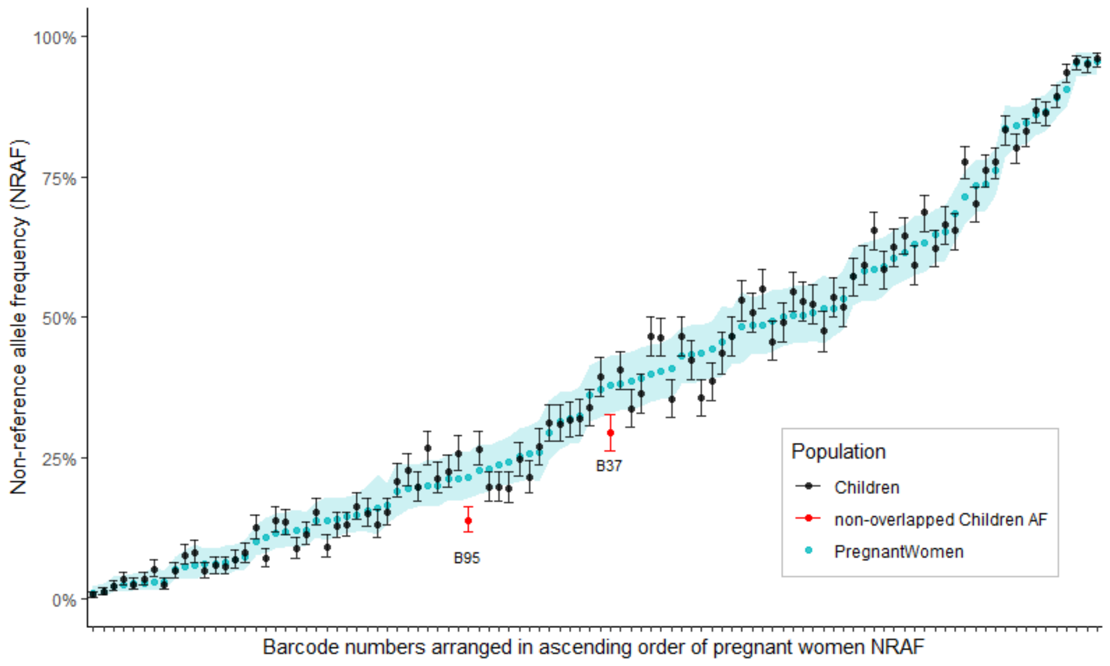

Supplementary Figure 4. Relationship between parasitaemia and genotype missingness

Values on the y-axis represent the percentage missingness and values on the x-axis the percentage parasitaemia. Each box represents the median and interquartile range of percentage parasitaemia by microscopy. The red dash line indicates 50% level of missingness.

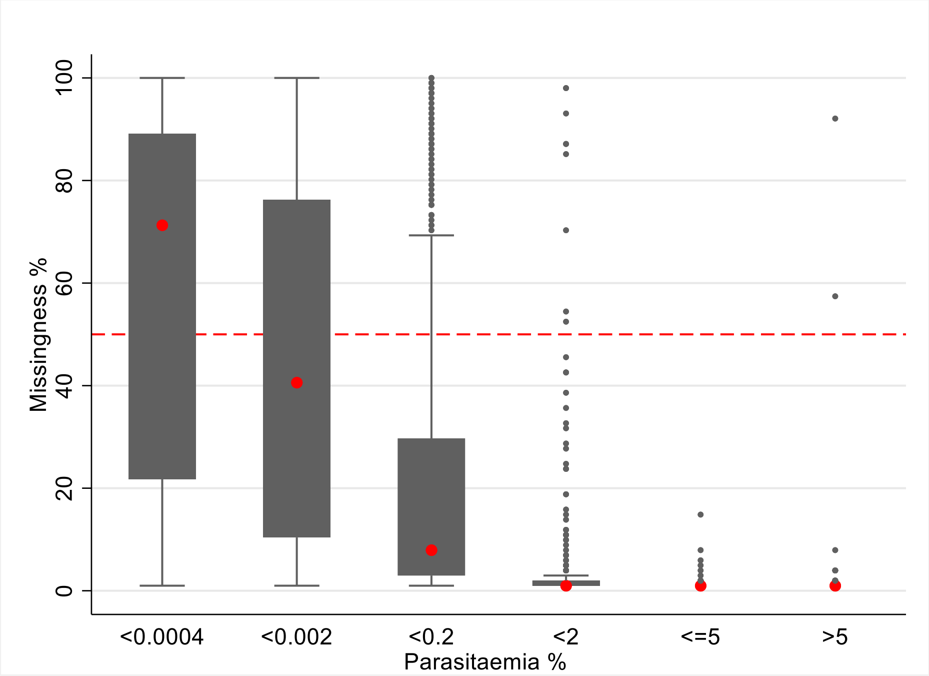

Supplementary Figure 5. Heterozygosity in the two cohorts

Each pink dot represents the percentage heterozygosity for one individual sample; each capped bar represents a population (pregnant women and children) with 95% percent Confidence Intervals.

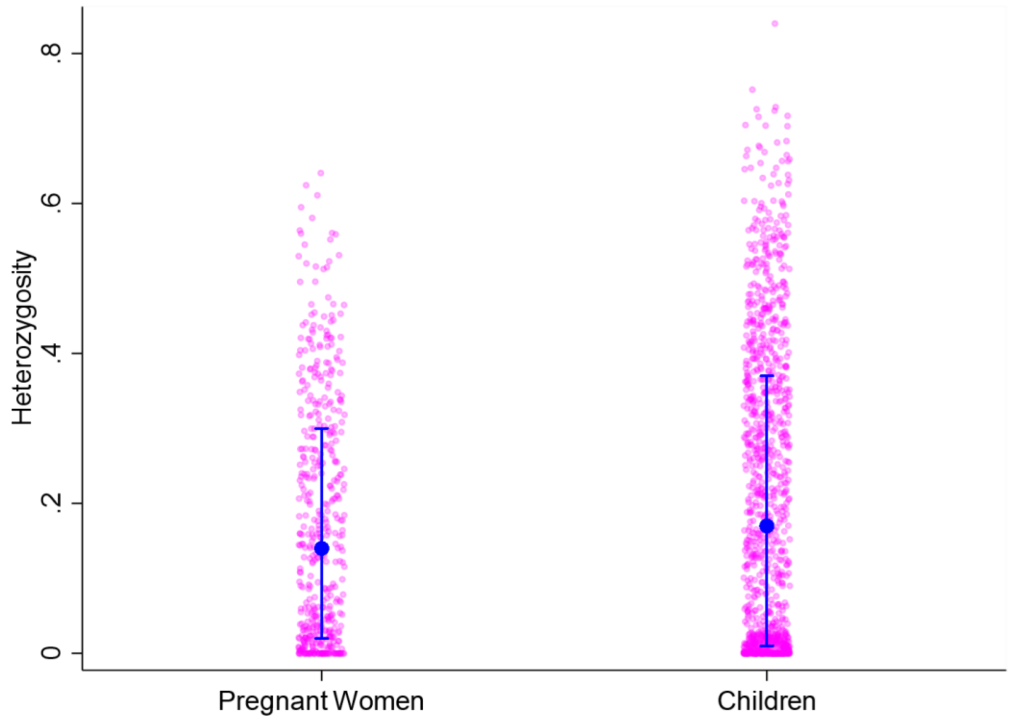

Supplement: Supplementary appendix 1 [file mmc1.pdf]
